# Diagnostic Accuracy of IgA Anti-Transglutaminase and IgG Anti-Deamidated Gliadin for Diagnosis of Celiac Disease in Children under Two Years of Age: A Systematic Review and Meta-Analysis

**DOI:** 10.3390/nu14010007

**Published:** 2021-12-21

**Authors:** Giulia N. Catassi, Alfredo Pulvirenti, Chiara Monachesi, Carlo Catassi, Elena Lionetti

**Affiliations:** 1 Department of Pediatrics, University La Sapienza, 00185 Rome, Italy; giulia.catassi@gmail.com; 2 Department of Mathematics and Bioinformatics, University of Catania, 95125 Catania, Italy; apulvirenti@dmi.unict.it; 3 Celiac Disease Research Laboratory, Marche Polytechnic University, 60123 Ancona, Italy; c.monachesi@pm.univpm.it; 4 Department of Pediatrics, Marche Polytechnic University, 60123 Ancona, Italy; c.catassi@univpm.it; 5 Center for Celiac Research, Mass General Hospital for Children, Boston, MA 02114, USA

**Keywords:** anti-deamidated gliadin peptide antibodies, anti-transglutaminase antibodies, celiac disease, children

## Abstract

The need of adding the determination of anti-deamidated gliadin peptide (DGP) IgG to anti-transglutaminase (TTG) IgA antibodies for diagnosis of celiac disease (CD) in children <2 years of age is controversial. We performed a systematic review and meta-analysis to evaluate, by head-to-head comparison, the diagnostic accuracy of TTG IgA and DGP IgG antibodies. We searched PubMed, MEDLINE, and Embase databases up to January 2021. The diagnostic reference was intestinal biopsy. We calculated the sensitivity and specificity of these tests and the odds ratio (OR) between the tests. Fifteen articles were eligible for the systematic review and ten were eligible for the meta-analysis. Sensitivity and specificity were 0.96 (95% confidence interval (CI), 0.91–0.98) and 0.96 (95% CI, 0.85–0.99) for DGP IgG and 0.93 (95% CI, 0.88–0.97) and 0.98 (95% CI, 0.96–0.99) for TTG IgA, respectively. TTG IgA specificity was significantly higher (OR 9.3 (95% CI, 2.3–37.49); *p* < 0.001) while the sensitivity of DGP IgG was higher without reaching statistical significance (OR: 0.6 (95% CI, 0.24–1.51); *p* = 0.28). Both the meta-analysis and the systematic review showed that some children with early CD are missed without the DGP IgG test. In children <2 years of age, TTG IgA is the best CD screening test; however, the addition of DGP IgG may increase the diagnostic sensitivity.

## 1. Introduction

Celiac disease (CD) is a multi-systemic disorder caused by gluten ingestion in genetically predisposed individuals that is characterized by an enteropathy of variable severity at the small intestinal biopsy [1]. With a population prevalence of 1–2%, CD is one of the most common life-long disorders. From a clinical perspective, CD is extremely variable and may affect subjects of any age. In so-called “typical” cases, the onset of CD is observed during the first two years of life, after months from the introduction of the gluten-free diet, usually with symptoms of intestinal malabsorption, particularly chronic diarrhea, loss of appetite, weight loss, and abdominal distention [1,2].

Several serological tests are available for CD diagnosis that detect antibodies directed against a fragment of the gluten antigen, anti-deamidated gliadin peptide (DGP), or self-antigens, anti-endomysial (EMA) and anti-tissue transglutaminase (TTG) antibodies, of both IgA and IgG classes [1]. In children, the widespread application of these tests has dramatically changed the CD diagnostic algorithm that was previously centered on the small intestinal biopsy. Based on extensive reviews of the literature, the major international diagnostic guidelines from the European Society for Pediatric Gastroenterology, Hepatology, and Nutrition (ESPGHAN) [3], the North American Society for Pediatric Gastroenterology, Hepatology, and Nutrition (NASPGHAN) [4], and the American College of Gastroenterology (ACG) [5] agree on recommending the TTG IgA antibody as the most cost-effective and reliable screening test to identify CD. Obtaining a serum IgA level at the initial testing is also recommended to identify those who have selective IgA deficiency and need an IgG-based test (TTG or DGP) [3,4,5]. However, the screening algorithm in children under two years of age is a controversial issue due to concerns on TTG IgA sensitivity in this age range [6,7,8,9]. ESPGHAN recommends initial testing with TTG IgA alone (plus total IgA), regardless of age [3], while NASPGHAN and ACG recommend that the TTG IgA should be combined with a DGP IgG determination to improve the diagnostic accuracy in children younger than two years [4,5].

The purpose of this study was to clarify whether DGP IgG should be added in testing children with suspected CD below two years of age. To this aim, we evaluated the diagnostic accuracy of TTG IgA and DGP IgG tests in young children by a “head-to-head” comparison, based on the meta-analysis and systematic review of available studies.

## 2. Materials and Methods

### 2.1. Protocol

Before review and meta-analysis, we developed a protocol, including eligibility criteria, search strategies, criteria for study selection, methods for extracting related data, and methods for assessing study quality and statistical methodology. The protocol was based on the PRISMA–DTA (preferred reporting items for systematic reviews and meta-analyses of diagnostic test-accuracy studies) guidelines [10].

### 2.2. Eligibility Criteria

All types of study design, such as comparative prospective studies, prospective cohort studies, retrospective case–control studies, and case reports, were considered for inclusion in the review. For the purpose of the meta-analysis, case reports were excluded. Only studies published in peer-reviewed journals and in English were considered, irrespective of main outcomes, date, or publication status. Children under two years of age with suspected CD were the focus of our search. The reference standard for the diagnosis of CD was intestinal biopsy. Our primary outcome measure was the diagnostic test accuracy of DGP IgG and TTG IgA (index tests). Only comparative primary studies (all patients performing both DGP IgG and TTG IgA) were included in the meta-analysis; comparative primary studies where some data were missing, were included only in the review. Prospective cohort studies, in which a series of patients from a given population were recruited and received the two index tests and a reference standard, with a long-term follow-up period, reporting the diagnostic accuracy of the two index tests at different ages, but not specifying the outcome at two years of age, were included only in the review.

### 2.3. Information Sources and Search 

A systematic electronic search of PubMed, MEDLINE, and Embase databases was conducted from their inception to January 2021. We combined the search terms for DGP antibodies (deamidated gliadin peptide) with keywords for CD, as follows “(celiac disease [MeSH Terms]) AND (deamidated gliadin peptide antibodies [MeSH Terms])”. The search was performed according to the PRISMA–S (PRISMA–S: an extension to the PRISMA statement for reporting literature searches in systematic reviews) guidelines [11]. In addition, reference lists of all included articles were searched.

### 2.4. Study Selection

Screening, eligibility assessment, and inclusion in the review and in the meta-analysis was performed independently in an unblinded standardized manner by two reviewers (E.L. and G.N.C.). Disagreements between reviewers were resolved by consensus.

### 2.5. Data Collection Process

We developed a data extraction sheet and pilot tested it on three randomly selected included studies, refining it accordingly. One author (G.N.C.) extracted the data from the included studies, and the second author (E.L.) checked the extracted data.

### 2.6. Definitions for Data Extraction

True positive was considered a diagnosis of CD, confirmed by the reference standard (a grade 2 or 3 lesion at the small-bowel biopsy according to the Marsh classification, on a scale of 0–3, with higher scores indicating villous atrophy) [12]. True negative was considered a diagnosis of non-CD, as shown by either (a) a grade 0 lesion at the small-bowel biopsy according to the Marsh classification or (b) negativity of all serological markers tested, when the biopsy was not available. Index tests (DGP IgG and TTG IgA) were considered positive if the numerical value was higher than the normal value. The pattern of clinical presentation of CD was defined as "classic" if the patient presented the classic picture of malabsorption (diarrhea, weight loss, and abdominal distension); “atypical”, in the presence of other clinical manifestations, including iron deficiency, short stature, aphtous stomatitis, and recurrent abdominal pain; "silent," in individuals apparently asymptomatic, diagnosed as part of a screening program [1]. The following information was extracted from each included study: (1) study characteristics (author and year of publication, study design); (2) characteristics of participants (number, gender, age at enrollment, age at CD diagnosis, and pattern of clinical presentation of CD); (3) true positives, true negatives, false positives, and false negatives for both DGP IgG and TTG IgA.

### 2.7. Risk of Bias and Applicability

To ascertain the quality of the studies we used the QUADAS (quality assessment of diagnostic accuracy studies)-2 tool [13], examining bias and applicability of the studies with respect to four separate domains, as follows: patient selection, index test, reference standard, and the flow and timing of patients through the study. No overall summary score was calculated, but for each domain, any concern with regard to bias and applicability were qualified as “low”, “high”, or “unclear”. In all cases, two authors (E.L. and G.N.C.) independently assessed the quality of the studies included, with any disagreement being resolved by discussion and consensus. Where necessary, study authors were contacted for additional information or for clarification of the study methods.

### 2.8. Diagnostic Accuracy Measures

We used the data from the two-by-two tables to calculate sensitivity and specificity with 95% confidence intervals (CI) for each study. We present individual study results graphically, by plotting the estimates of sensitivity and specificity in both forest plots and the receiver operating characteristic (ROC) space. To compare the sensitivity and the specificity of the two index tests, we calculated the odds ratio (OR) for each study, between the following: (a) the number of false negatives for DGP and the number of false negatives for TTG, and (b) the number of false positives for DGP and the number of false positives for TTG. 

### 2.9. Meta-Analysis

We performed the meta-analysis by using the random-effects model with the meta-package of the R system. The Mantel–Haenszel inverse variance was used for pooling. As a measure of heterogeneity, we computed the statistic I^2^, defined as the percentage of total variance across studies, which was attributable to heterogeneity rather than chance. Measures were computed on subsamples with low heterogeneity values [14].

## 3. Results

### 3.1. Study Selection

**Figure 1:**
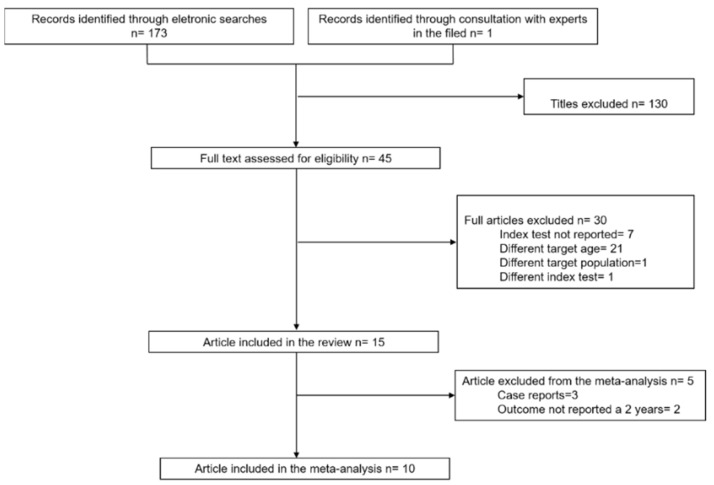
PRISMA flow diagram of study selection.

Figure 1 presents a flow diagram that summarizes the results of the literature search. A total of 174 articles were identified. Of them 45 articles were deemed potentially eligible and were full-text evaluated. A total of 15 articles met the eligibility criteria for the systematic review and were included [15,16,17,18,19,20,21,22,23,24,25,26,27,28,29]; of them, 10 were also included in the meta-analysis [15,16,17,18,19,20,21,22,23,24].

### 3.2. Study Characteristics

Table 1 provides broad details of the studies included in the systematic review and meta-analysis [15,16,17,18,19,20,21,22,23,24,25,26,27,28,29]. We did not find any pediatric systematic review nor meta-analysis. Ten studies were included in both the systematic review and meta-analysis [15,16,17,18,19,20,21,22,23,24]. Of them, five were comparative prospective studies aimed to verify the diagnostic performance of DGP IgG as compared with TTG IgA for CD diagnosis in children under two years of age [15,16,17,18,19]; they all included the following: (a) a group of children referred to a tertiary care center for suspected CD and presenting with classical symptoms—they were tested for both DGP IgG and TTG IgA, and the diagnosis was confirmed or excluded by the reference standard (intestinal biopsy); (b) a control group of healthy children, negative for serological markers of CD, age, and sex-matched to the studied patients. Of these prospective studies, one [16] was excluded from the analysis of sensitivity of TTG IgA and from the comparison of sensitivity between the two index tests because it included only children with high serum levels of DGP IgG but normal values of TTG IgA in the group of suspected CD; additionally, it did not give the results of the overall sample of children with suspected CD who were screened for both tests. The study of Monzani [17] was excluded from the analysis of specificity of the index tests because it did not report the results of both tests in children under two years of age in the control group. Five studies were comparative retrospective studies, aimed at evaluating diagnostic performance of DGP IgG compared with TTG IgA for CD diagnosis in pediatric age [20,21,22,23,24]. They included consecutive pediatric patients tested for TTG IgA and DGP IgG, who were referred to a tertiary care center and underwent intestinal biopsy because of suspected CD; therefore, CD diagnosis was confirmed or excluded based on the reference standard. The study of Hojsak was excluded from the analysis of specificity of the index tests because it did not report the results of both tests in children without CD [24]. For all these retrospective studies, data on children under two years of age were extracted from the overall results. 

For the purpose of the systematic review, five more studies were included, i.e., three case reports [25,26,27] and two prospective longitudinal cohort studies assessing the timing of DGP IgG and TTG IgA antibodies appearance in children who developed CD [28,29].

Overall, the systematic review included 7553 participants. Studies included in the meta-analysis involved 6000 participants. Most included studies recruited participants from Italy. All studies included participants attending a tertiary healthcare facility.

The risk of selective reporting bias was rated high for one study [16] because it included children with suspected CD and high serum levels of DGP IgG, but normal values of TTG IgA, and did not report results on the overall sample of children with suspected CD who were tested for both tests. This study was therefore excluded from the sensitivity analysis of TTG IgA, and from the comparison of sensitivity between tests. For all included studies, the risk of bias was rated low with respect to the index tests, while it was unclear, with respect to the reference standard, because it was not reported whether the pathologist who interpreted the biopsy (reference standard) was always the same person during the study and whether he/she was blinded to serology (index tests). The risk of bias with respect to flow and timing was rated low for all included studies. There were no applicability concerns. None of the articles included in the meta-analysis reported a power calculation to determine the population size necessary to answer the research question.

### 3.3. Results of Individual Studies

**Figure 2:**
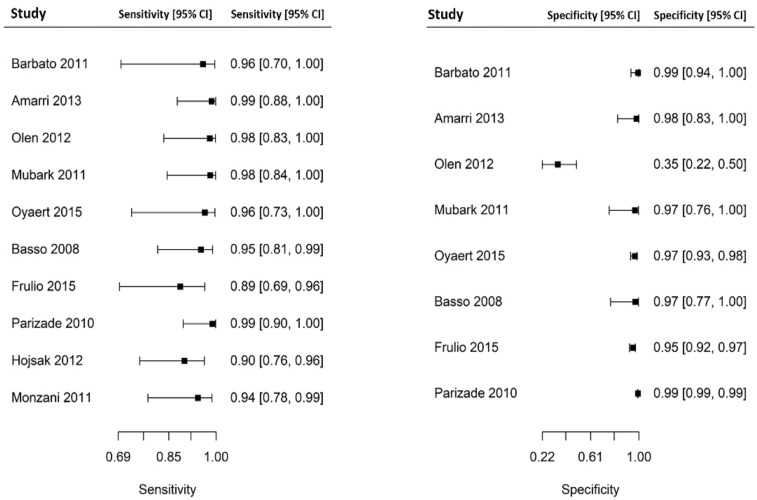
Forest plot for sensitivity and specificity of immunoglobulin G anti-deamidated gliadin peptide antibody (DGP IgG) determination.

**Figure 3:**
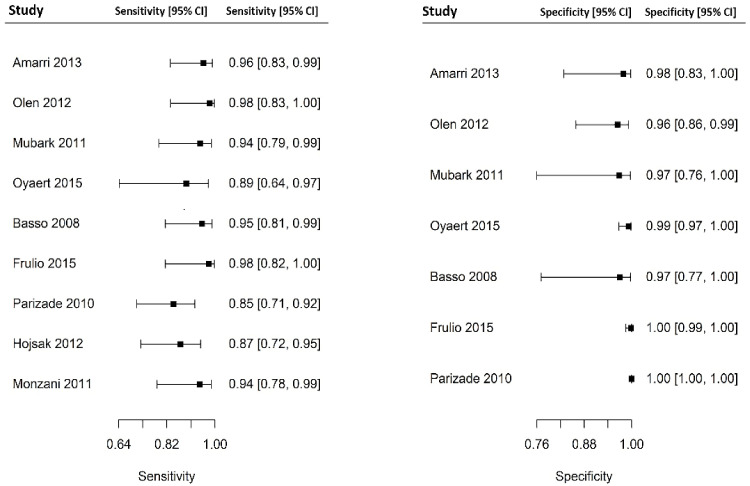
Forest plot for sensitivity and specificity of immunoglobulin A anti-tissue transglutaminase antibody (TTG IgA) determination.

**Figure 4:**
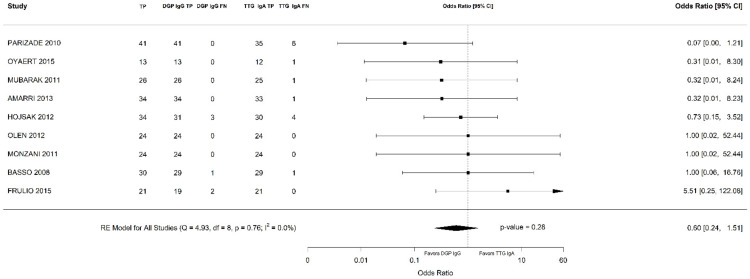
Forest plot of direct comparison of sensitivity between immunoglobulin G anti-deamidated gliadin peptide antibody (DGP IgG) and immunoglobulin A anti-tissue transglutaminase antibody (TTG IgA) determination. Only comparative primary studies reporting the number of total true positives (TP) and false negatives (FN) for DGP IgG and TTG IgA are included.

**Figure 5:**
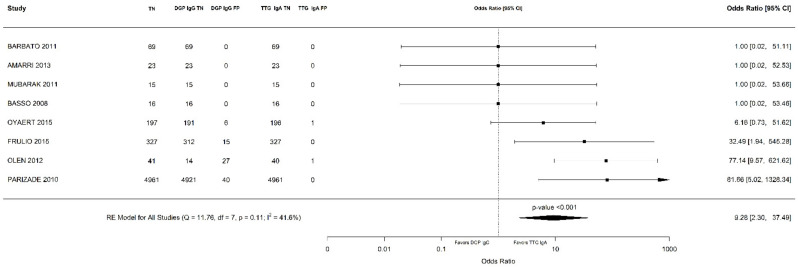
Forest plot of direct comparison of specificity between immunoglobulin G anti-deamidated gliadin peptide antibody (DGP IgG) and immunoglobulin A anti-tissue transglutaminase antibody (TTG IgA) determination. Only comparative primary studies reporting the number of total true negatives (TN) and false positives (FP) for DGP IgG and TTG IgA are included.

Out of 6000 children included in the meta-analysis, 5930 were included in the comparison of sensitivity between the two index tests; 247 had a confirmed diagnosis of CD by intestinal biopsy and were therefore considered true positives. Of these, 241 tested positive for DGP IgG as compared with 233 children testing positive for TTG IgA (6 children were DGP IgG false negative, and 14 children were TTG IgA false negative). A total of 5968 children were included in the comparison of specificity between the tests; of them, 5649 were true negatives. Of these, 5561 tested negative for DGP IgG as compared with 5647 children testing negative for TTG IgA. The summary sensitivity and specificity of DGP IgG were 0.96 (95% CI, 0.91–0.98) and 0.96 (95% CI, 0.85–0.99), respectively. The summary sensitivity and specificity of TTG IgA were 0.93 (95% CI, 0.88–0.97) and 0.98 (95% CI, 0.96–0.99), respectively. Figure 2 and Figure 3 show forest plots for sensitivity and specificity of DGP IgG and TTG IgA, respectively. Supplementary Figures S1 and S2 show the coupled summary ROC curves for sensitivity and specificity of DGP IgG and TTG IgA, respectively. Based on these findings, in a hypothetical cohort of 1000 children tested for CD, among whom 500 actually have the disease, TTG IgA and DGP IgG will give 33 and 22 false negatives (children with CD for whom the diagnosis of CD will be missed), respectively. Direct comparisons between the two index tests were based on 9 studies for sensitivity and 8 studies for specificity, and are shown in Figure 4 and Figure 5, respectively. No substantial heterogeneity was observed. Although the sensitivity of DGP IgG for CD diagnosis was higher, the OR for the sensitivity of DGP IgG versus TTG IgA was not significant (O.R: 0.6 (95% CI, 0.24–1.51); *p* = 0.3), while the OR for specificity was significantly higher for TTG IgA as compared with DGP IgG (OR: 9.3 (95% CI, 2.3–37.49); *p* ≤ 0.001). 

Overall, out of 7553 children included in the systematic review, 319 had a confirmed diagnosis of CD; of them, there were 28 false negatives for TTG IgA and 5 were false negatives for DGP IgG. True negatives were 7234; of them, 112 were false positives for TTG IgA and 207 were false positives for DGP IgG.

In detail, the study of Olen [22] reported the following: (a) two young celiac children who had been biopsied because of major gastro-intestinal symptoms, who were TTG IgA negative, but were not tested for DGP, showing a poor performance of TTG IgA; (b) four non-CD, DGP IgG-positive children who were not tested for TTG IgA. These data have not been included in the meta-analysis because only one index test was performed. The study of Barbato [16] reported 11 children with classic symptoms of CD who were TTG IgA negative but DGP IgG positive, with a final diagnosis of CD confirmed by intestinal biopsy. These data were not included in the comparison of sensitivity between the tests, as already explained.

The systematic review also showed that, in longitudinal prospective studies performed in children at genetic risk of CD followed from birth by serological screening, DGP antibodies often precede TTG seroconversion, suggesting that frequent monitoring of both TTG and DGP antibodies may allow earlier detection of CD in genetically susceptible children [28,29]. In detail, Liu and college [28] analyzed sera, prospectively, from children with an increased risk for CD participating in the Denver study and found that DGP antibodies appeared earlier than TTG IgA in 9 out of 50 children with CD, suggesting that the measurement of these antibodies may be useful for earlier screening in CD. Lammi et al. [29] screened 291 newborns at genetic risk for CD and found that all of the children who developed CD were DGP IgG positive at the time of TTG IgA seroconversion, and in over half of the cases, DGP IgG positivity preceded TTG seroconversion a median 12 months earlier. The 3 selected case reports, respectively, described the following: (a) an 18 months child with CD diagnosed because of classic symptoms, and typical intestinal biopsy findings, who showed a high level of DGP IgG and negative TTG IgA [25]; (b) an 8 months child with CD diagnosed because of classic symptoms and positive intestinal biopsy, who showed high level of both DGP IgA and IgG and negative TTG IgA [26]; (c) a 23 months child with CD and peripheral neuropathy who presented with high levels of DGP IgG (16 × the normal level) and intermediate levels of TTG IgA (4 × the normal level) [27].

## 4. Discussion

The principal findings of this systematic review and meta-analysis are as follows: (a) the diagnostic accuracy of both TTG IgA and DGP IgG in children under two years of age is high; (b) the sensitivity of DGP IgG and TTG IgA is similar, while TTG IgA is superior to DGP IgG as far as the specificity is concerned; (c) some children with CD are missed if DGP IgG is not performed at the initial testing. 

Serologic testing for the diagnosis of CD has evolved in the last few decades, and, currently, the recommended initial screening test for CD is TTG IgA [1,2]. However, despite the high sensitivity of TTG IgA in adults and older children, there has historically been hesitation about the application of this test in younger children under two years of age, as highlighted in a recent editorial on this issue [30]. Several studies using either anti-native gliadin antibodies or DGP IgG and TTG IgA antibodies suggested that TTG IgA performs less well in this age group [6,7,8,9]. These concerns underpinned recommendations to obtain complementary serologic testing, especially DGP IgG, to increase screening and diagnostic sensitivity of TTG IgA in children below two years of age. The NASPGHAN and the ACG guidelines on the diagnosis of CD recommend the inclusion of the DGP IgG test with TTG IgA testing in children under two years of age [4,5]. The 2012 ESPGHAN guidelines recommended measuring DGP antibodies as an additional test in children who are negative for other CD-specific antibodies but in whom clinical symptoms raise a strong suspicion of CD, especially if they are younger than two years old [31,32]. However, in the 2020 revised ESPGHAN guidelines, this statement has been removed, and it is recommended that in subjects with normal serum IgA values for their age, only TTG IgA should be used as the initial serological test, regardless of age [3]. This recommendation is based on the assumption that adding DGP testing to TTG IgA testing seldom improves sensitivity, while specificity markedly decreases. 

To the best of our knowledge, this the first study to evaluate the diagnostic accuracy of DGP IgG in a head-to-head comparison with TTG IgA in children under two years of age, by a systematic review of all available evidence and with a meta-analytical approach. By a head-to-head comparison, we found that the sensitivity of DGP IgG was slightly higher but not statistically superior to TTG IgA, probably due to the small sample size of the few available studies included in the meta-analysis. The systematic review of studies not included in the meta-analysis confirmed that some children with early CD are missed if DGP IgG is not performed. These patients are typically young children with classical symptoms of CD with negative TTG IgA, but positive DGP IgG. Since a short delay in diagnosis may be responsible for life-threatening manifestations in this age-group, as suggested by one case report included in this review [25], the addition of DGP IgG in these symptomatic cases is strongly advisable in our opinion. The presence of DGP IgG and the negativity of TTG IgA may be linked to the immature immunological response of children under two years of age, who do not yet produce autoantibodies (TTG), while producing antibodies against the external antigens (DGP and AGA). As the disease progresses, antibodies are eventually produced against both gliadin and TTG. Full expression of EMA and TTG antibodies generally occurs after 2–3 years of age [9,30]. Indeed, we also found, by the systematic review of longitudinal cohort studies, that DGP IgG often precedes TTG IgA seroconversion, suggesting that DGP IgG antibodies determination may allow earlier detection of CD. However, the meta-analysis also showed that the specificity of DGP IgG is significantly lower compared with TTG IgA, confirming that always adding DGP IgG to TTG IgA determination may lead to an excessive and unhelpful number of small-bowel biopsies in DGP IgG-positive, TTG IgA-negative children. Therefore, in our opinion, a reasonable compromise could be the combined determination of TTG IgA and DGP IgG in the subset of young children with a high clinical suspicion of CD to avoid a potentially dangerous delayed or missed diagnosis. Future studies should also evaluate the diagnostic accuracy of adding DGP IgA to TTG IgA determination in young children.

The weakness of our review and meta-analysis is that few studies, so far, primarily compared the diagnostic accuracy of DGP IgG and TTG IgA in children under two years of age, and none of the available studies had performed a power calculation to determine the population size necessary to answer the research question. Moreover, in none of the available studies was the diagnosis of CD in cases with negative autoantibodies confirmed by a gluten challenge, as recommended in the latest guidelines.

In conclusion, this review and meta-analysis show that adding DGP IgG determination to TTG IgA may improve the diagnostic accuracy of CD detection in children under two years of age, especially in those with a strong clinical suspicion of CD. A revision of the ESPGHAN clinical guidelines could be appropriate in view of these findings, in the effort to standardize the recommendations worldwide. Further evidence from large prospective studies evaluating the diagnostic accuracy of DGP IgG and TTG IgA in a head-to-head comparison in this age range is advisable.

## Figures and Tables

**Table 1:** Characteristics of included studies.

Publication	Study Type and Location	Total No. Included	Gender (%Female)	Age	Population	Target Condition	Reference Test	Index Tests	TP	TN	TP DGP IgG	TP TTG IgA	TN DGP IgG	TN TTG IgA
Parizade 2010 [15]	Prospective controlled, Israel	5002	NR	<2 yr	200 symptomatic children; 4802 healthy children	CD	Intestinal biopsy	DGP IgG and TTG IgA	41	4961	41/41	35/41	4912/4961	4961/4961
Barbato 2011 [16]	Prospective controlled, Italy	80	43	4–24 mo	40 children with classic symptoms of CD and with DGP IgG positive and TTG IgA negative; 40 healthy children	CD	Intestinal biopsy	DGP IgG and TTG IgA	11	69	11/11	0/11	69/69	69/69
Monzani 2011 [17]	Prospective, controlled, Italy	130	NR	<2 yr	24 children with classic symptoms of CD; 106 healthy children	CD	Intestinal biopsy	DGP IgG and TTG IgA	24	106	24/24	24/24	NR	NR
Amarri 2013 [18]	Prospective controlled, Italy	57	62	<2 yr	34 children with classic symptoms of CD; 23 healthy children	CD	Intestinal biopsy	DGP IgG and TTG IgA	34	23	34/34	33/34	23/23	23/23
Oyaert 2015 [19]	Prospective controlled, Belgium	210	44	<2 yr	13 symptomatic children; 197 healthy children	CD	Intestinal biopsy	DGP IgG and TTG IgA	13	197	13/13	12/13	191/197	196/197
Basso 2008 [20]	Retrospective, Italy	46	NR	<2 yr	46 symptomatic children undergoing intestinal biopsy	CD	Intestinal biopsy	DGP IgG and TTG IgA	30	16	29/30	29/30	16/16	16/16
Mubarak 2011 [21]	Retrospective, The Netherlands	41	NR	<2 yr	41 symptomatic children undergoing intestinal biopsy	CD	Intestinal biopsy	DGP IgG and TTG IgA	26	15	26/26	25/26	15/15	15/15
Olen 2012 [22]	Retrospective, Sweden	71	NR	<2 yr	71 symptomatic children undergoing intestinal biopsy	CD	Intestinal biopsy	65 children tested for DGP IgG and TTG IgA; 2 tested for TTG IgA only; 4 tested for DGP IgG only)	26 (24 tested for both index tests; 2 tested only for TTG IgA).	45 (41 tested for both index tests; 4 tested only for DGP IgG)	24/24	24/26	14/45	40/41
Frulio 2015 [23]	Retrospective, Italy	348	49	6–24 yr	348 symptomatic children undergoing intestinal biopsy	CD	Intestinal biopsy	DGP IgG and TTG IgA	21	327	19/21	21/21	312/327	327/327
Hojsak 2012 [24]	Retrospective, Israel	31	46	<2 yr	31 symptomatic children undergoing intestinal biopsy	CD	Intestinal biopsy	DGP IgG and TTG IgA	31	NR	31/31	30/30	NR	NR
Catassi 2020 [25]	Case report, Italy	1	100	18 mo	1 child presenting with CD crisis	CD	Intestinal biopsy	DGP IgG and TTG IgA	1	NA	1/1	0	NA	NA
Arigliani 2017 [26]	Case report, Italy	1	0	8 mo	1 child presenting with failure to thrive, constipation, and developmental delay	CD	Intestinal biopsy	DGP IgG and TTG IgA	1	NA	1/1	0	NA	NA
Pacitto 2017 [27]	Case report, Italy	1	0	23 mo	1 child presenting with malabsorption syndrome and peripheral neuropathy	CD	Intestinal biopsy	DGP IgG and TTG IgA	1	NA	1/1	1/1	NA	NA
Liu 2015 [28]	Longitudinal cohort, USA	1243	NR	0.5–17 yr	1243 newborn at genetic risk of CD screened with both index tests	CD	Intestinal biopsy	DGP IgG and TTG IgA	50	1193	50/50	50/50	NR	NR
Lammi 2016 [29]	Longitudinal cohort, Finland	291	NR	6–48 mo	291 newborn at genetic risk of CD screened with both index tests	CD	Intestinal biopsy	DGP IgG and TTG IgA	9	282	9/9	9/9	NR	NR

R: not reported; yr: years; mo: months; CD: celiac disease; DGP IgG: immunoglobulin G anti-deamidated gliadin peptide antibody; TTG IgA: immunoglobulin A anti-tissue transglutaminase antibody; TP: true positive; TN: true negative; NA: not applicable.

## Data Availability

The data presented in this study are available on request from the corresponding author.

## Supplementary Materials

The following are available online at https://www.mdpi.com/article/10.3390/nu14010007/s1, Figure S1: Summary receiver operating curve of combined sensitivity and specificity of immunoglobulin G anti-deamidated gliadin peptide antibodies (DGP IgG), Figure S2: Summary receiver operating curve of combined sensitivity and specificity of immunoglobulin A anti-tissue transglutaminase antibodies (TTG IgA).
