# Supplementary material for: Diagnostic Accuracy of IgA Anti-Transglutaminase and IgG Anti-Deamidated Gliadin for Diagnosis of Celiac Disease in Children under Two Years of Age: A Systematic Review and Meta-Analysis
Source: Nutrients. 2021 Dec 21;14(1):7. doi: 10.3390/nu14010007 (PMC8746847; doi:10.3390/nu14010007)
Supplement: Supplementary file 1 [file nutrients-14-00007-s001.zip › nutrients-1477108-supplementary.pdf]

Review

Diagnostic Accuracy of Antibodies against Transglutaminase and Deamidated Gliadin for Diagnosis of Celiac Disease in Children under Two Years of Age: A Systematic Review and Meta-Analysis

Supplementary material

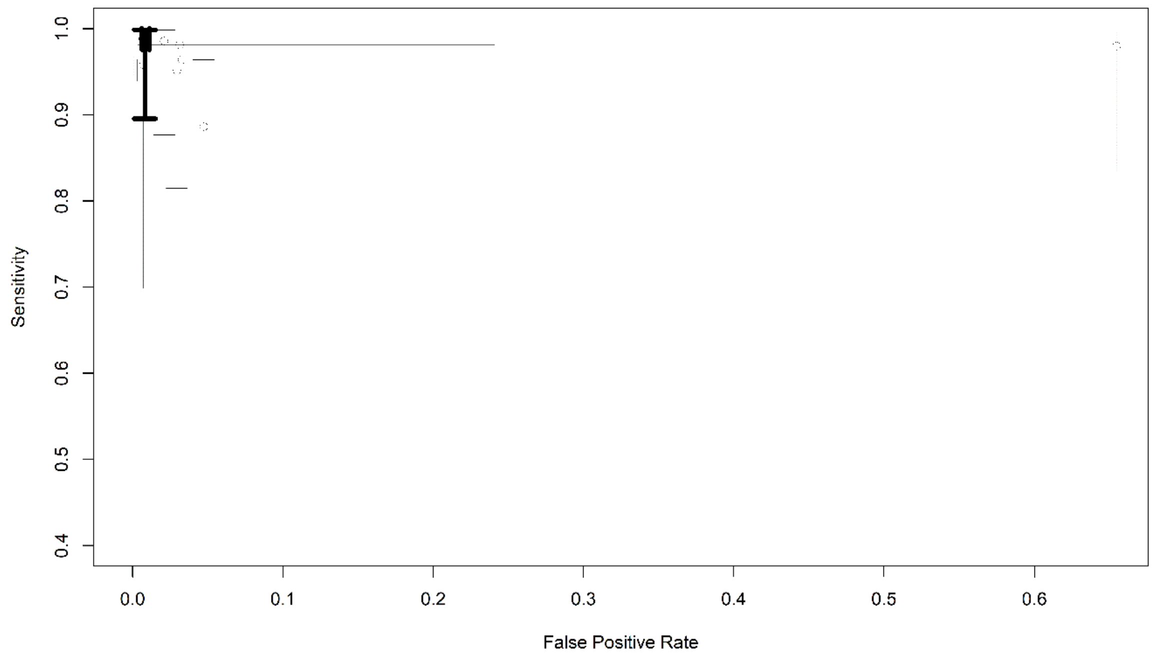

Supplementary figure S1. Summary receiver operating curve of combined sensitivity and specificity of immunoglobulin G anti-deamidated gliadin peptide antibodies (DGP IgG).

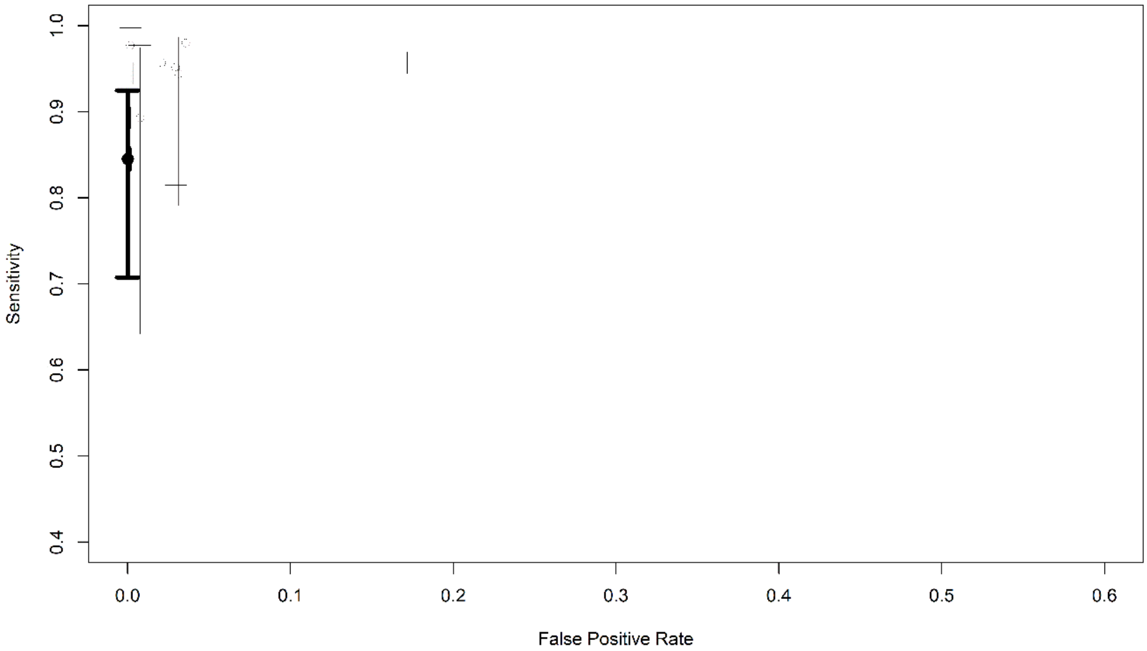

Supplementary figure S2. Summary receiver operating curve of combined sensitivity and specificity of immunoglobulin A anti-tissue transglutaminase antibodies (TTG IgA).
